# Influence of chronic kidney disease on early clinical outcomes after off-pump coronary artery bypass grafting

**DOI:** 10.1186/s13019-020-01245-5

**Published:** 2020-07-29

**Authors:** Xihui Li, Siyu Zhang, Feng Xiao

**Affiliations:** Department of Cardiac Surgery, Peking University First Hospital, 8 Xishiku Street, Beijing, 100034 People’s Republic of China

**Keywords:** Chronic kidney disease, Coronary artery bypass grafting, Off-pump

## Abstract

**Background:**

Patients with chronic kidney disease (CKD) have a high incidence of coronary heart disease, which is the leading cause of death in these patients. Coronary artery bypass grafting (CABG) significantly increases short-term mortality and decreases long-term mortality in patients with CKD compared with percutaneous coronary intervention (PCI). The effect of CKD on the early outcomes of off-pump CABG is not well-studied. We aimed to investigate the effect of CKD on early postoperative mortality and complications following off-pump CABG.

**Methods:**

We retrospectively analyzed preoperative baseline and surgery data for 1173 patients undergoing off-pump CABG from January 2010 to December 2017 in the Department of Cardiac Surgery, Peking University First Hospital. Outpatient follow-up was performed until 30 days postoperatively. Patients with estimated glomerular filtration rates calculated according to the Chronic Kidney Disease Epidemiology Collaboration equation of ≥60 mL/min/1.73 m^2^ were assigned to the normal renal function group (normal group, *n* = 924), and those with a rate < 60 mL/min/1.73 m^2^ were assigned to the CKD group (CKD group, *n* = 249).

**Results:**

Patients in the CKD group were seriously ill with multiple complications, and postoperative 30-day mortality and complication rates were significantly higher than those in the normal group. In the logistic regression analysis, after correcting for common confounding factors, namely sex, age, and left ventricular ejection fraction, preoperative CKD was a risk factor for postoperative acute kidney injury, perioperative myocardial infarction, gastrointestinal bleeding, secondary tracheal intubation, stroke, chest wound infection, prolonged mechanical ventilation (≥ 24 h), prolonged intensive care unit stay (≥ 72 h), prolonged length of stay (≥ 14 d), dialysis requirement, and postoperative death within 30 days.

**Conclusions:**

Patients with CKD had more preoperative complications, and their postoperative 30-day mortality and complication rates after off-pump CABG were significantly higher than those of patients with normal renal function. For CABG patients with CKD, the risk of surgery should be assessed carefully, and comprehensive measures should be taken to strengthen perioperative management, with an aim to reduce complications and mortality and improve surgical outcomes.

## Background

Patients with chronic kidney disease (CKD) have a high incidence of coronary heart disease [1], which is the leading cause of death in these patients. Concurrent CKD and percutaneous coronary intervention (PCI) or coronary artery bypass grafting (CABG) increases mortality in patients undergoing revascularization [2, 3]. However, CABG significantly increases short-term mortality and decreases long-term mortality in patients with CKD compared with PCI [4]. Currently, off-pump CABG is the main surgical procedure in mainland China, and accounts for approximately 95% of the total number of CABG procedures in our department. The effect of CKD on the early outcomes of off-pump CABG is not well-studied. We retrospectively studied the early outcomes of 1173 patients undergoing off-pump CABG from January 2010 to December 2017 in the Department of Cardiac Surgery, Peking University First Hospital.

## Methods

### Study design and subjects

Inclusion criteria: consecutive, first-time, off-pump CABG patients. Exclusion criteria: patients with preoperative acute renal insufficiency, preoperative dialysis dependence, and intraoperative transfer to extracorporeal circulation. We used the Chronic Kidney Disease Epidemiology Collaboration (CKD-EPI) equation to calculate estimated glomerular filtration rates (eGFR) [5]. Patients with eGFR_CKD-EPI_ ≥ 60 mL/min/1.73 m^2^ were assigned to the normal renal function group (normal group), and those with GFR_CKD-EPI_ < 60 mL/min/1.73 m^2^ were assigned to the CKD group. We collected data for patients during hospitalization and from their postoperative outpatient follow-up records within 30 days, for statistical analysis. The clinical observation outcomes were stroke, prolonged mechanical ventilation (≥ 24 h), reintubation, redo for bleeding, perioperative myocardial infarction (PMI), upper gastrointestinal bleeding (UGH), new atrial fibrillation, acute respiratory distress syndrome (ARDS), low cardiac output syndrome (LCOS), perioperative use of intra-aortic balloon pump (IABP), chest wound infection, acute kidney injury (AKI), prolonged intensive care unit (ICU) stay (≥ 72 h), prolonged length of stay (≥ 14 d), dialysis replacement, and postoperative death within 30 days. PMI was defined as increased cardiac troponin I (CTNI) values to > 10 times baseline values, or new pathological Q waves. ARDS was defined as partial pressure of arterial oxygen to fraction of inspired oxygen (PaO_2_/FiO_2_) ≤ 300 mmHg and positive end-expiratory pressure (PEEP) or continuous positive airway pressure (CPAP) ≥ 5 cmH_2_O, and classic chest radiographic changes. LCOS was defined as cardiac index < 2.0 L/min/m^2^, with sufficient blood volume and systolic pressure < 90 mmHg. AKI was defined as a sudden decrease in renal function within 48 h after operation, absolute value of serum creatinine increased by > 26 μmol/L, or serum creatinine increased by > 50% or urine volume decreased to < 0.5 mL/kg/h for more than 6 h.

All patients were treated with routine tracheal inhalation and intravenous anesthesia, implantation of a Swan–Ganz floating catheter, median sternotomy, and off-pump coronary artery bypass grafting. The left internal mammary artery and/or great saphenous vein and/or non-dominant radial artery were used as graft vessels. The distal graft vessels were anastomosed with an Octopus® tissue stabilizer (Medtronic, Inc., Minneapolis, MN, USA), and the proximal graft vessels were anastomosed with side wall forceps after partial aortic occlusion. If calcification of the ascending aorta was serious, proximal anastomosis was used to anastomose the proximal part of the graft vessel.

### Statistical analysis

We used SPSS version 21.0 (IBM Corp., Armonk, NY, USA) to analyze the data, and the *Χ*^*2*^ test or Fisher’s exact test to compare the enumeration data between the two groups. Measurement data were analyzed using the t test. We performed logistic regression to analyze the effect of preoperative renal function status on postoperative mortality and complications. Odds ratios (OR) and 95% confidence intervals (CI) were calculated after univariate regression analysis and after correcting for common confounding factors, such as sex, age, and left ventricular ejection fraction (LVEF). Two-sided *p* < 0.05 was considered statistically significant.

## Results

Preoperative patients’ baseline clinical data are shown in Table 1. Female sex and advanced age, with hypertension, diabetes, lower LVEF, and lower hemoglobin level were more common in the CKD group (*p* < 0.001) compared with the normal group, as were preoperative stroke, atrial fibrillation, and lower albumin level (*p* < 0.05).

**Table 1:** Preoperative patients’ baseline clinical data between the two groups

	Normal Group (*n* = 924)	CKD Group (*n* = 249)	*t/Χ*^*2*^	*P*
Age (ys)	63.5 ± 9.3	69.8 ± 9.2	9.618	< 0.001†
Female sex	228 (24.7)	117 (47)	47.035	< 0.001†
Hypertension	607 (65.7)	208 (83.5)	29.444	< 0.001†
Diabetes	353 (38.2)	130 (52.2)	15.884	< 0.001†
Stroke	195 (21.1)	71 (28.5)	6.142	0.013†
Hyperlipemia	389 (42.1)	118 (47.4)	2.237	0.135
COPD	25 (2.7)	8 (3.2)	0.185	0.667
AF	40 (4.3)	21 (8.4)	6.704	0.01†
Emergency	18 (1.9)	7 (2.8)	0.701	0.403
Diseased coronary vessels				
Triple	602 (65.1)	155 (62.3)	1.569	0.210
Left main	33 (3.6)	10 (4.0)	0.110	0.740
Left main + Triple	217 (23.5)	70 (28.1)	2.246	0.134
Others (single or two)	72 (7.8)	14 (5.6)	1.359	0.244
LVEF(%)	61.1 ± 13.2	57.4 ± 13.2	3.579	< 0.001†
Hemoglobin(g/L)	135.0 ± 15.4	121.9 ± 18.7	10.216	< 0.001†
Albumin(g/L)	40.1 ± 13.8	37.9 ± 4.2	2.484	0.013†

*COPD* Chronic obstructive pulmonary disease, *AF* Atrial fibrillation, *LVEF* Left ventricular ejection fraction

†Significant difference

Surgery and follow-up data within 30 days are shown in Table 2. Complication rates in the CKD group and postoperative death within 30 days were significantly higher than those in the normal group. The results of the logistic regression analysis of the influence of preoperative renal function status on postoperative death and complications after CABG are shown in Table 3. Preoperative CKD was a risk factor for postoperative complications and postoperative 30-day all-cause mortality (OR: 5.309; *p* < 0.001). After correcting for the common confounding factors of sex, age, and LVEF, preoperative CKD was no longer a risk factor for LCOS (OR: 1.444; *p* = 0.449), perioperative IABP use (OR: 1.617; *p* = 0.054), postoperative new atrial fibrillation (OR: 1.244; *p* = 0.240), or prolonged mechanical ventilation time (OR: 1.248; *p* = 0.216).

**Table 2:** Surgery and follow-up data within 30 days between the two groups

	Normal Group (*n* = 924)	CKD Group (*n* = 249)	*t/Χ*^*2*^	*P*
AKI	135 (14.6)	59 (23.7)	11.727	< 0.001†
PMI	165 (17.9)	55 (22,1)	2.305	0.129
UGH	12 (1.3)	11 (4.4)	–	0.004†
AF	155 (16.8)	64 (25.7)	10.296	< 0.001†
LCOS	63 (6.8)	32 (12.9)	9.719	0.002†
IABP	63 (6.8)	40 (16.1)	20.935	< 0.001†
Redo for bleeding	10 (1.1)	4 (1.6)	–	0.512
Reintubation	13 (1.4)	13 (5.2)	13.164	< 0.001†
ARDS	93 (10.1)	35 (14.1)	3.152	0.076
Wound infection	18 (2.0)	13 (5.2)	8.146	0.004†
Stroke	14 (1.5)	11 (4.4)	7.905	0.005†
Ventilation time ≥ 24 h	176 (19.1)	66 (26.5)	6.536	0.011†
ICU stay≥72 h	273 (29.6)	111 (44.6)	20.031	< 0.001†
LOS ≥ 14d	345 (37.3)	147 (59.0)	37.924	< 0.001†
Dialysis replcement	2 (0.2)	9 (3.6)	–	< 0.001†
Death	8 (0.9)	11 (4.4)	–	< 0.001†

*AKI* Acute kidney injury, *PMI* Perioperative myocardial infarction, *UGH* Upper gastrointestinal hemorrhage, *AF* Atrial fibrillation, *LCOS* Low cardiac output syndrome, *IABP* Intra-aortic balloon pump, *ARDS* Acute respiratory distress syndrome, *LOS* Length of stay

†Significant difference

**Table 3:** Results of the logistic regression analysis

	Unadjusted				Adjusted			
	OR	95%	CI	*p*	OR	95%	CI	*p*
AKI	1.815	1.286	2.561	0.001†	1.550	1.071	2.244	0.02†
PMI	1.304	0.925	1.839	0.130	1.237	0.856	1.789	0.257
UGH	3.509	1.529	8.051	0.003†	2.716	1.102	6.691	0.030†
AF	1.716	1.231	2.393	0.001†	1.244	03864	1.789	0.240
LCOS	2.025	1.290	3.178	0.002†	1.444	0.877	2.378	0.149
IABP	2.616	1.712	3.997	0.001†	1.617	0.992	2.636	0.054
Redo for bleeding	1.492	0.464	4.799	0.502	1.989	0.561	7.046	0.287
Reintubation	3.860	1.766	8.438	0.001†	2.514	1.074	5.885	0.034†
ARDS	1.456	0.960	2.209	0.077	1.152	0.736	1.802	0.537
Wound infection	2.770	1.338	5.733	0.006†	2.586	1.171	5.714	0.019†
Stroke	3.001	1.345	6.695	0.007†	2.789	1.165	6.676	0.021
Ventilation≥24 h	1.527	1.102	2.115	0.011†	1.248	0.879	1.773	0.216
ICU ≥ 72 h	1.915	1.437	2.552	0.001†	1.658	1.218	2.258	< 0.001†
LOS ≥ 14d	2.419	1.818	3.218	0.001†	1.828	1.348	2.478	< 0.001†
Dialysis replacement	17.287	3.711	80.538	0.001†	23.153	4.534	118.238	< 0.001†
Death	5.309	2.112	13.345	0.001†	3.424	1.251	9.374	0.017†

*AKI* Acute kidney injury, *PMI* Perioperative myocardial infarction, *UGH* Upper gastrointestinal hemorrhage, *AF* Atrial fibrillation, *LCOS* Low cardiac output syndrome, *IABP* Intra-aortic balloon pump, *ARDS* Acute respiratory distress syndrome, *LOS* length of stay

†Significant difference

## Discussion

An epidemiological survey in 2012 indicated that the incidence of CKD in China was 10.8%, and that the estimated number of existing CKD patients was 119.5 million [6]. The risk of coronary heart disease is significantly increased in patients with CKD. Inflammatory reactions, oxidative stress, impaired endothelial cell function, coronary artery calcification, hyperhomocysteinemia, immune suppression, and other mechanisms participate in accelerating the progression of atherosclerosis, which leads to a poor prognosis in this population. Coronary heart disease-related complications, including myocardial infarction and heart failure, are the main causes of death [7]. In patients with CKD, lipid metabolism disorders, mainly hypertriglyceridemia, are risk factors for complications related to coronary heart disease. This risk factor results in approximately 12% of people with stage 3 or more advanced CKD also having coronary heart disease, compared with 5% of people with normal renal function [8]. Patients with CKD also often have associated bone mineral metabolism disorders such as hypocalcemia and hyperphosphatemia, as well as secondary hyperparathyroidism, which also accelerate systemic atherosclerosis and vascular calcification [9]. Currently, the number of patients with CKD in mainland China is very high, and many more patients with coronary heart disease will require CABG in the future.

For patients with CKD with severe coronary heart disease and not receiving dialysis, CABG significantly reduces mortality, reinfarction, and revascularization rates compared with PCI and drug therapy [10, 11]. For low-risk patients, CABG does not improve survival compared with PCI and oral medications, but significantly improves survival for high-risk patients. CABG increases the incidence of postoperative renal failure [12]. In a nonrandomized study prospectively analyzing patients’ data, 2108 patients with CKD and with drug-eluting stents underwent PCI (*n* = 1165) and CABG (*n* = 943), with a mean follow-up of 41.4 months. Although there were no significant differences in all-cause death, stroke, or myocardial infarction rates between the two groups, the revascularization rate was significantly higher in the PCI group (adjusted hazard ratio: 4.72; 95% CI: 3.20–6.96; *p* < 0.001) [13]. A meta-analysis of 29,246 patients enrolled in 11 studies showed that CABG was associated with lower long-term all-cause mortality, lower cardiac mortality, lower incidence of myocardial infarction and revascularization, and fewer major cardiac and cerebrovascular adverse events compared with drug-eluting stents in revascularization for coronary heart disease in patients with multivessel disease and CKD [14]. For patients with type 2 diabetes, CKD and stable coronary heart disease, CABG and optimal drug therapy did not reduce the incidence of major cardiac and cerebrovascular adverse events, but significantly reduced the proportion of patients requiring revascularization [15]. Therefore, CABG has a significant advantage over PCI and drug therapy in patients with coronary heart disease and CKD.

CABG is the key to protecting renal function and improving postoperative survival rates in patients with CKD; however, whether to select off-pump or on-pump CABG is controversial. According to current research, off-pump CABG may have more advantages because it has a protective effect on renal function perioperatively in avoiding cardiopulmonary bypass, and reduces complications related to allogeneic blood transfusion, postoperative thoracotomy hemostasis, acute kidney injury, and respiratory problems [16]. However, in the CORONARY study, no significant difference in the effect of the two surgical procedures was seen regarding renal function at the 1-year follow-up, and off-pump CABG had no long-term renal protective effect [17]. Ueki et al. reviewed data from 38,051 patients undergoing CABG alone and assigned patients to separate groups according to renal function. The results showed that in the mild renal insufficiency group, there was no significant reduction in the risk of death from off-pump CABG compared with the on-pump CABG group. In the moderate to severe renal insufficiency group, compared with on-pump CABG, off-pump CABG significantly reduced surgical mortality and the risk of requiring postoperative dialysis, in patients with severe renal insufficiency [18].

Off-pump CABG is the main surgical method in our department. In this study, patients with CKD were mainly older women, and were more often complicated with hypertension, diabetes, stroke, atrial fibrillation, hypoproteinemia, anemia, and lower LVEF. Postoperative 30-day mortality (*p* < 0.001) and complications in the CKD group were significantly higher than in the normal group in our study. Logistic regression analysis showed that preoperative CKD increased postoperative complications and postoperative 30-day mortality. After correcting for sex, age, and LVEF, preoperative CKD remained a risk factor for the following complications: AKI, gastrointestinal bleeding, secondary endotracheal intubation, stroke, chest wound infection, prolonged ICU stay, prolonged length of stay, dialysis replacement, and postoperative death within 30 days. Therefore, even with off-pump CABG, postoperative complications and 30-day mortality in patients with CKD remained significantly higher than in those with normal renal function.

Preoperative CKD and worse renal function is associated with increased hospital stay and costs. Creatinine clearance rates decreasing from 80 mL/min to 60 mL/min, 40 mL/min, and 20 mL/min result in total hospital expenses increasing by 10, 20, and 30%, respectively; the incidence and mortality related to dialysis also increases [19]. Therefore, it is necessary to strengthen perioperative management and develop comprehensive strategies for high-risk patients, to improve prognosis and reduce complications and mortality. We suggest: 1. Preoperative assessment of high-risk patients with CKD is conducive to rational allocation of medical resources and targeted prevention and management. More severe preoperative CKD is associated with higher surgical mortality. A retrospective analysis of 483,914 patients undergoing CABG alone showed that the operative mortality rate was 1.3% in patients with normal renal function and increased to 9.3% in those with severe renal insufficiency (GFR < 30 mL/min/1.73 m^2^) not on dialysis [20]. Conversely, for every 10 mL/min/1.73 m^2^ increase in eGFR, the risk of death decreases by 20% [21]. The use of the CKD-EPI equation to calculate eGFR for grouping patients according to CKD severity is also a good predictor of postoperative complications and mortality [22]. Charytan et al. suggested that factors such as repeat cardiac surgery, stroke, cardiogenic shock, emergency surgery, and composite valve surgery should be included in the preoperative risk assessment to identify high-risk patients for surgery; however, the specificity and sensitivity of these factors require verification [23]. 2. Active control of complications, such as controlling hypertension, correcting anemia, and rational treatment of peripheral artery disease is important. Patients with CKD have a high incidence of hypertension, which may lead to or result from CKD. Hypertension aggravates CKD, which then increases the difficulty of controlling hypertension [24]. Anemia is also a common manifestation with renal insufficiency, and renal dysfunction leads to decreased erythropoietin secretion and anemia. Both anemia and CKD can predict myocardial ischemia in patients with coronary heart disease; the severity of the anemia is associated with the degree of myocardial ischemia [25]. 3. New targeted drug therapies such as small-dose human atrial natriuretic peptide injections perioperatively during on-pump CABG can improve perioperative cardiac and renal function and reduce the incidence of cardiac events and new dialysis requirement [26]. However, the role of human atrial natriuretic peptide injections in off-pump CABG must be verified.

## Conclusion

Patients with CKD have significantly more preoperative complications, and higher 30-day mortality and complication rates after off-pump CABG than patients with normal renal function. For patients with CKD scheduled for CABG, the risk of surgery should be assessed carefully, and comprehensive measures should be taken to strengthen perioperative management, with an aim to reduce complications and mortality and improve surgical outcomes.

## Data Availability

Please contact the corresponding author for data requests.
